# Preclinical Research in McArdle Disease: A Review of Research Models and Therapeutic Strategies

**DOI:** 10.3390/genes13010074

**Published:** 2021-12-28

**Authors:** Mónica Villarreal-Salazar, Astrid Brull, Gisela Nogales-Gadea, Antoni L. Andreu, Miguel A. Martín, Joaquín Arenas, Alfredo Santalla, Alejandro Lucia, John Vissing, Thomas O. Krag, Tomàs Pinós

**Affiliations:** 1 Mitochondrial and Neuromuscular Disorders Unit, Vall d’Hebron Institut de Recerca, Universitat Autònoma de Barcelona, 08035 Barcelona, Spain; monicaazucena.villarreal@e-campus.uab.cat; 2 Centro de Investigación Biomédica en Red de Enfermedades Raras (CIBERER), 28029 Madrid, Spain; mamcasanueva.imas12@h12o.es (M.A.M.); joaquin.arenas@salud.madrid.org (J.A.); 3 Neuromuscular and Neurogenetic Disorders of Childhood Section, National Institute of Neurological Disorders and Stroke, National Institutes of Health, Bethesda, MD 20892, USA; astrid.brullcanagueral@nih.gov; 4 Grup de Recerca en Malalties Neuromusculars i Neuropediàtriques, Department of Neurosciences, Institut d’Investigacio en Ciencies de la Salut Germans Trias i Pujol i Campus Can Ruti, Universitat Autònoma de Barcelona, 08916 Barcelona, Spain; gnogales@igtp.cat; 5 EATRIS, European Infrastructure for Translational Medicine, 1081 HZ Amsterdam, The Netherlands; toniandreu@eatris.eu; 6 Mitochondrial and Neuromuscular Diseases Laboratory, 12 de Octubre Hospital Research Institute (i+12), 28041 Madrid, Spain; 7 Department of Sport Sciences, Universidad Pablo de Olavide, 41013 Sevilla, Spain; asanher@upo.es; 8 Faculty of Sport Sciences, European University, 28670 Madrid, Spain; alejandro.lucia@universidadeuropea.es; 9 Copenhagen Neuromuscular Center, Department of Neurology, Rigshospitalet, University of Copenhagen, DK-2100 Copenhagen, Denmark; John.Vissing@regionh.dk (J.V.); thomas.krag@regionh.dk (T.O.K.)

**Keywords:** McArdle disease, glycogen, glycogen phosphorylase, research models, treatments

## Abstract

McArdle disease is an autosomal recessive disorder of muscle glycogen metabolism caused by pathogenic mutations in the *PYGM* gene, which encodes the skeletal muscle-specific isoform of glycogen phosphorylase. Clinical symptoms are mainly characterized by transient acute “crises” of early fatigue, myalgia and contractures, which can be accompanied by rhabdomyolysis. Owing to the difficulty of performing mechanistic studies in patients that often rely on invasive techniques, preclinical models have been used for decades, thereby contributing to gain insight into the pathophysiology and pathobiology of human diseases. In the present work, we describe the existing in vitro and in vivo preclinical models for McArdle disease and review the insights these models have provided. In addition, despite presenting some differences with the typical patient’s phenotype, these models allow for a deep study of the different features of the disease while representing a necessary preclinical step to assess the efficacy and safety of possible treatments before they are tested in patients.

## 1. Introduction

McArdle disease [glycogen storage disease (GSD) type V or myophosphorylase deficiency (OMIM^®^ #232600; http://www.omim.org, accessed on 1 December 2021)] is an autosomal recessive disorder of muscle glycogen metabolism that was first described by Brian McArdle in 1951 [1]. The estimated prevalence of this condition is ~1/100,000 people, with both sexes equally affected [2,3]. McArdle disease is caused by inherited deficiency of myophosphorylase, the skeletal muscle-specific isoform of glycogen phosphorylase (GP-MM), with preservation of the other two glycogen phosphorylase isoforms, the liver (GP-LL) and brain glycogen phosphorylase (GP-BB); as such, this defect essentially leads to myopathy with no alterations of glycogen metabolism reported in other tissues [4]. However, the fact that GP-MM expression has been detected not only in skeletal muscle—or in organs containing skeletal muscle tissue such as the tongue, glands and esophagus—but also in different parts of the brain (e.g., cerebellum), lymphoid tissues, lymphocytes, granulocytes, retina, kidney, male reproductive organs or adipose tissue [5,6,7,8,9], deserves attention and should be the subject of further studies.

GP-MM is encoded by the *PYGM* gene located on chromosome 11q13 [10]. A total of 206 pathogenic mutations have been described in this gene [11,12,13,14,15,16,17,18,19,20,21,22,23,24,25], with the most prevalent in the Caucasian population (allele frequency ~50%) being the nonsense c.148C>T p.(R50*) variant [16,26,27,28,29,30,31,32,33]. There is no association between disease phenotype and *PYGM* genotype, as the vast majority of mutations generally cause a complete loss of GP-MM activity [26,31,33,34,35,36]. GP-MM initiates glycogenolysis in skeletal muscle, degrading muscle glycogen by removing (1,4)-alfa-glucosyl units from the branches and releasing glucose-1-phosphate. Thus, because GP-MM is absent―or virtually absent―in patients with McArdle disease, these individuals are unable to obtain energy from muscle glycogen stores leading to abnormal accumulation of subsarcolemmal vacuoles of glycogen [26,37].

Clinical symptoms are mainly characterized by transient acute “crises” of early fatigue, myalgia and contractures, which can be accompanied by rhabdomyolysis. The latter is reflected by very high circulating levels (at least one order of magnitude above normal) of proteins released from damaged muscle, such as creatine kinase (CK) or aspartate aminotransferase (a transaminase that is also found in the liver), and sometimes by myoglobinuria, with the latter visually reflected as the presence of “dark urine” [26,37]. Massive rhabdomyolysis could eventually lead to acute renal failure [26,37]. Although the clinical manifestation is typically moderate and appears during childhood or early adulthood, some severe cases have been reported with clinical onset at neonatal age [26,37]. Exercise intolerance is triggered by static or isometric exercises imposing low metabolic demands but high mechanical loads on relatively low muscle mass (e.g., lifting or carrying weights), as well as by dynamic exercise involving larger muscle mass (e.g., walking and especially brisk walking or running). During isometric exercise, muscles largely rely on anaerobic glycogenolysis for contraction, while during endurance dynamic exercises, such as brisk walking, muscles relay largely on aerobic pathways, including not only fat but also glycogen oxidation [37]. On the other hand, patients typically experience the so-called “second wind” phenomenon after 7–10 min of dynamic exercise, an almost pathognomic feature (which has also been observed in the proximal glycolytic defect, phosphoglucomutase 1 deficiency [38]) characterized by a drop in heart rate and perceived exertion, so that exercise (e.g., brisk walking) which appeared strenuous and painful during the first minutes now becomes more tolerable [26,37]. The first few minutes of exercise indeed act as a warm-up (stimulating vaso-dilatation), after which more circulating free fatty acids as well as glucose are readily available to working muscle fibers that can oxidize these substrates, leading to attenuation of early exercise intolerance [26,39].

The effect of impaired muscle glycogenolysis (e.g., due to depletion of muscle glycogen depots during endurance exercise) on exercise and metabolic adaptation in humans has been well described, but the underlying pathophysiological mechanisms have yet to be disclosed. In this regard, whether naturally or generated in the laboratory, animal models often have the advantage of allowing the researcher to perform mechanistic studies that cannot always be performed in humans for obvious ethical reasons while providing insights into the pathophysiology of a given disorder [40]. In the following, we will review the animal and cell culture models available for McArdle disease and describe the insights these models have provided.

## 2. Animal Models

### 2.1. Charolais Cattle

The first described spontaneous animal model for McArdle disease was a Charolais cattle identified in Davis, California [41] (Table 1). It was initially depicted in a 7-week-old female calf that presented with a 2-day history of recumbency after forced exercise (round up from a large pasture) [41]. The calf was unable to rise or support its own weight and had stiff muscles. Electrolyte abnormalities included hyperkalemia, hyperphosphatemia and hypocalcemia. Serum CK and aspartate aminotransferase levels were significantly increased, reflecting muscle damage. Extensive myonecrosis and calcification were detected throughout the whole body and numerous small thrombi were found histologically [41]. Shortly after that, a second calf from the same herd showed exercise intolerance, as well as episodes of collapse and recumbency with forced exercise. Further examination of the herd revealed four other cattle with exercise intolerance [41]. Histological examination of the semitendinosus/semimembranosus muscles of the affected calves showed the presence of subsarcolemmal vacuoles, muscle fibers with central nuclei and absence of myophosphorylase staining. Biochemistry analyses revealed null myophosphorylase activity together with muscle glycogen concentrations that were on average 1.6 times higher than in controls [41]. Interestingly, in one calf, 50% of the muscle fibers were necrotic, depleted of glycogen and contained degenerated mitochondria [41]. To analyze the cause of the disease in this breed of cattle, cDNA of GP-MM was sequenced and cloned, with the results revealing the missense mutation c.1468C>T(p.R490W) in the *PYGM* gene. This arginine residue is highly conserved in the three GP isoforms of several different species and is adjacent to pyridoxal phosphate binding sites [42].

### 2.2. Ovine Model

A second spontaneous animal model for McArdle disease was identified in a Merino sheep flock of Western Australia, which since then has been maintained at the Murdoch University Veterinary [43,46] (Table 1). Animals presented with exercise intolerance and muscle biopsies showed lack of myophosphorylase and excess of glycogen. They presented an A to G substitution in the 3′ acceptor splice site of intron 19 of the *PYGM* gene (c.2380-1G>A). This mutation resulted in the activation of a cryptic splice site in exon 20, thereby producing a frame-shift in the reading frame, with the subsequent premature termination of the GP-MM protein and removal of the last 31 amino acids [43].

### 2.3. Mouse Model

The aforementioned models of McArdle disease are of little practical use owing to the intrinsic difficulty of working with big animals. A knock-in mouse model of McArdle disease was therefore generated by our group [44] (Table 1). This model carries two copies of the most common pathogenic mutation in Caucasian patients, the stop codon mutation (p.R50*) in exon 1 of *PYGM* gene [11]. McArdle mice recapitulate the majority of phenotype traits that are characteristic of patients. Thus, their muscles are devoid of GP-MM protein and activity, and present massive muscle glycogen stores. Another hallmark of the disease, high serum CK levels accompanied by myoglobinuria following vigorous exercise, is also present. The key findings in the McArdle mouse model are summarized in the sections below.

#### 2.3.1. Body Mass

McArdle mice (p.R50*/p.R50*) do not show differences in body mass compared to their wild-type (WT, p.R50R/p.R50R) counterparts, a finding replicated in both young (8-week-old) [40] and adult (20-week-old) mice [47]. Additionally, no differences in heart-body ratios have been observed between genotypes, suggesting no cardiomyopathy associated with McArdle disease [47].

#### 2.3.2. Exercise Intolerance

A very poor performance has been observed in McArdle mice in the treadmill and wire grip tests, as indicators of tolerance to endurance dynamic and isometric exercise, respectively. As for treadmill tests, after an initial warm-up period (5 min at a speed of 5 cm s^−1^), the treadmill speed was increased to 15 cm s^−1^ for 5 min and subsequently by 5 cm s^−1^ every 5 min until exhaustion. Two different treadmill inclinations were evaluated (0% and 25%). McArdle mice showed significantly lower maximal endurance capacity compared to WT mice (~37% and ~29% of WT values at 0% and 25% inclination, respectively) [40,44]. Regarding the wire grip test, all WT mice were able to stay on the grip the maximum time established as per experimental protocol (i.e., 180 s), whereas none of the McArdle mice were able to stay more than 34 s [44]. In turn, nearly one-third of HTZ mice did not reach the maximum time, but all stayed more than 41 s, which again reflected the importance of retaining full GP-MM activity [44].

#### 2.3.3. Low Survival Rate

A longitudinal study with 139 matings and more than 2000 offspring showed intrinsic difficulties associated with the management of the McArdle mouse colony basically due to high levels of perinatal and post-weaning mortality [48]. In this regard, perinatal death has also been observed in GSD-0b, Ia, Ib, IV, VII and XV mouse models [49], indicating that glycogen might play a crucial role in energy supply during the neonatal period. On the other hand, perinatal death has never been observed in McArdle disease patients.

#### 2.3.4. Decreased Glucose and Lactate but Increased CK and Ammonia Blood Levels

Basal blood glucose and lactate levels from 8-week-old McArdle mice are lower than in WT mice, whereas ammonia levels are higher [39]. As expected, blood CK levels are higher in McArdle mice than in WT mice [40]. In patients, in turn, basal glucose and lactate levels are similar to those found in control individuals [50], but CK and ammonia levels are also higher [2,51,52]. Higher ammonia levels might reflect, at least partly, a metabolic deficit due to blocked glycogenolysis—that is, reliance on the purine nucleotide cycle to produce ATP [53].

#### 2.3.5. Massive Glycogen Accumulation Disrupts Myofibril Structure

Massive glycogen accumulation has been reported in muscles from 8-week-old McArdle mice compared to WT controls (i.e., 86-, 46- and 29-fold higher levels in *extensor digitorum longus* (EDL), *gastrocnemius* and *soleus* muscle, respectively) [40]. In turn, muscle glycogen levels in McArdle patients are “only” approximately two times higher than normal, with the enormous glycogen depots in McArdle mice causing structural alterations across and along the muscle fiber that are rarely seen in patients (see also Section 2.3.6). Indeed, muscles from McArdle mice show fibers in disarray, huge variability in size and diameter, large intrafiber voids filled with cytosolic glycogen granules and also the presence of internal nuclei as a result of ongoing degeneration/regeneration cycles [47,54]. Electronic microscopy images reflect that the huge amount of glycogen depots within the fibers cause not only a replacement of the myofibrils with glycogen, but also alterations in the cellular ultrastructure [47]. Thus, glycogen accumulation causes myofibrils to tear, change direction and split from adjacent fibers. As a result, T-tubules appear misaligned to myofibrils, and sarcoplasmic reticula are considerably enlarged [47]. Despite the major loss of structure in the affected fibers, fibro-fatty acid replacement has not been found in any of the muscles analyzed. Additionally, trichrome staining does not reveal ragged red fibers, suggesting that glycogen accumulation does not lead to formation of aggregates of abnormal mitochondria [47]. However, these findings do not translate into a reduction of the length, mass or cross-sectional areas of muscle fibers, at least in several muscles [soleus, EDL, *tibialis anterior* (TA), quadriceps and diaphragm] from 20-week-old McArdle mice [47]. Treadmill exercise does not further exacerbate these histopathological features (i.e., no differences between exercised and non-exercised muscles) [54]. With regard to the muscle contractile function, both twitch and tetanic forces are significantly lower in the soleus and EDL of McArdle mice than in WT mice [31]. Of note, contractile force is further reduced in the EDL (a predominantly fast-twitch muscle) compared to the soleus (a muscle with a slow-twitch phenotype), while an increase in fatigability is only seen in the EDL [47]. Interestingly, progressive muscle degeneration during disease progression does not seem to be associated with an increase in glycogen content [48].

#### 2.3.6. Fiber Type-Specific Degeneration

Massive glycogen accumulation causing muscle damage seems to occur mainly in fibers containing myosin heavy chains (MHC) type IIA, IIX and a mix of I/IIA and IIA/IIX, all of which are predominantly found in glycolytic, fast-twitch muscles (vs. muscles with a more oxidative, slow-twitch phenotype) [47]. In contrast, low glycogen accumulation and less fiber degeneration is observed in pure type I slow oxidative fibers, as well as in fibers containing a mix of MHC IIX/IIB or pure MHC IIB fibers, which make up the majority of fibers in highly glycolytic muscles [40,47,48]. A comparative histological study of the oxidative soleus muscle and three glycolytic muscles, i.e., the TA, EDL and quadriceps, revealed that only the latter was spared from devastating glycogen accumulation [47]. In this regard, when the percentage of centrally nucleated fibers was analyzed and compared between soleus, EDL, TA and quadriceps from 20-week-old McArdle mice, a significant difference among muscle types was found; TA and soleus presented the highest levels (~15%) of centrally nucleated fibers, while EDL and quadriceps had half that level [47]. Furthermore, acute levels of muscle fiber regeneration (measured as percentage of embryonic MHC positive fibers) are significantly higher in TA than in quadriceps of McArdle mice [47]. Interestingly, in contrast to what has been observed in patients or in the sheep model, regeneration-related expression of *Pygb* and/or *Pygl* has not been found in TA or quadriceps of McArdle mice [47]. The observed histopathological differences between TA and quadriceps in McArdle mice might be explained by the higher proportion of IIB fibers in the latter. In the case of the soleus, although this muscle is abundant in type I fibers, it seems to be as affected as TA and EDL muscles due to its content of mixed or pure IIA and IIX MHC fibers [47]. In turn, muscles from patients appear to have fewer mixed fiber types than McArdle mice and glycogen accumulation does not differ across the different fiber types. Thus, the inability to break down glycogen leads to more structural degeneration in the muscles of McArdle mice than in patients [47]. Of note, no major changes in the relative distribution of the different muscle fiber types (based on MHC profiles) have been reported in patients with McArdle disease or in McArdle mice compared to their controls [48,55].

#### 2.3.7. Differential Metabolic Responses among Muscles

In a first study, the protein levels of the enzymes directly involved in glycogen synthesis (glycogen branching enzyme (GBE), muscle glycogen synthase (GS-MM)) and degradation (glycogen debranching enzyme (GDE) and GP-MM) were analyzed in both oxidative (soleus) and glycolytic muscles (gastrocnemius and EDL) of 8-week-old WT and McArdle mice [40]. GBE protein levels were twofold higher in the latter, although no differences were reported for the soleus muscle [40] (Table 2). These findings would suggest a compensatory mechanism in the gastrocnemius and EDL in order to accommodate higher amounts of glycogen in more tightly packed granules—with such adaptive response not needed in the soleus, a muscle that shows naturally higher levels of GBE than EDL in rodents [40,56]. In fact, it has been suggested that glycogen granules might have more ramifications and be more densely packed in oxidative fibers than in glycolytic fibers [56]. In addition, GS-MM protein levels are much lower in McArdle mice, which is accompanied by 2–3-fold increases in its phosphorylated form, thereby suggesting an inhibition of glycogen synthesis in both oxidative and glycolytic muscles in order to prevent further deleterious accumulation of glycogen [40] (Table 2). With regard to glycogen degradation, GDE protein levels are lower in the soleus of McArdle mice than in their WT counterparts, but no differences have been found in the EDL and gastrocnemius [40] (Table 2). This result along with the lower expression of GP-MM and GDE enzymes in the soleus of WT mice in comparison to more glycolytic muscles, suggest that oxidative muscles are not as dependent on glycogen catabolism to ensure a proper function as glycolytic muscles, and thus, they might be less affected by GP-MM deficiency. As expected, no detectable levels of the GP-MM protein have been reported in any of the muscles analyzed in McArdle mice; however, in HTZ mice there is a differential decrease in GP-MM levels among these muscles compared to WT (~60% in the soleus and ~35% in both gastrocnemius and EDL) [40]. These results were further complemented in a second study analyzing the glycogen/glucose turnover signaling pathways in four different muscles (soleus, EDL, TA and quadriceps) of 16–20-week-old McArdle mice [57]. In contrast with the results obtained in 8-week-old animals, no significant changes in GS-MM protein levels were observed in the soleus, EDL and TA of McArdle mice, while a slight increase was observed in the quadriceps [57]. Furthermore, no differences in the phosphorylation status of the GS-MM were detected in the soleus and EDL of McArdle mice, although a significant increase was found in TA and quadriceps [57] (Table 2). In addition, GS-MM activity in the absence of glucose-6-phosphate was almost null in the quadriceps of McArdle mice [57]. Further differences in the levels of glycogen metabolism proteins between 8- and 20-week-old McArdle mice were observed, as increases in GBE protein levels were only found in the quadriceps of McArdle mice [57]. With regard to glucose uptake, phospho-Thr 172-AMPK (pAMPK) was only upregulated in the high glycolytic muscles (TA and quadriceps, especially the latter) [57,58] (Table 2). Moreover, quadriceps also presented increases in phospho-Ser 237-TBC1 domain family member 1 (pTBC1D1) and Glut4 that were not observed in TA muscle [57] (Table 2). The fact that the proportion of IIB fibers in McArdle mice is twice higher in the quadriceps than in TA might explain why the quadriceps muscle is more dependent on contraction-induced glucose uptake for direct metabolism, thereby sparing glucose use to form glycogen and, thus, to contribute to further glycogen accumulation [47]. In this regard, it has been observed that the dependency on glucose from glycogen is strikingly different between TA and quadriceps, as TA has seven times higher GP-MM protein levels than quadriceps [47]. However, all these observations have been reported in non-exercised mice; after treadmill performance, pAMPK protein levels were also increased in the EDL muscle. Moreover, there was an increment in the sarcolemma localization of Glut 4 protein in the TA of McArdle mice in comparison to non-exercised mice. Additionally, treadmill performance also induced an upregulation of hexokinase 2 in TA, EDL and soleus muscles that was not observed under resting conditions [54]. Finally, with regard to the oxidative metabolism, only the biceps *femoris* muscle has been analyzed so far in McArdle mice, showing a decrease in citrate synthase activity but no significant changes in the activity of different oxidative phosphorylation (OXPHOS) complexes [58] (see also Section 2.3.8).

Overall, the aforementioned results suggest that in the quadriceps of McArdle mice, there is an upregulation of the contraction-induced glucose uptake pathway, with an increase in pAMPK, pTBC1D1 and Glut 4 protein levels along with a decrease in glycogen synthesis. On the other hand, in the vastus lateralis of McArdle patients, glucose uptake occurs through the activation of the insulin dependent pathway, and subsequent direct disposal of glucose through the glycolytic pathway as increased levels of phospho-Ser 473-Akt, Glut4, hexokinase II and fructose-6-phosphate kinase have been reported, along with the activation of epinephrine stimulated inhibition of glycogen synthesis [57] (Table 2).

#### 2.3.8. No Changes in Oxidative Phosphorylation Proteins Levels and Activity

No significant changes in NDUFB8, SDHB, CORE2, CVα and CS protein levels have been found in the quadriceps between 8-week-old WT and McArdle mice [58]. Additionally, no differences in OXPHOS complex activities (CI, CII, CIII, CIV, CI+III and CII+III) were observed between genotypes. However, CS activity was significantly lower in McArdle mice [58]. In McArdle disease patients, the muscle oxidative capacity is severely reduced, as observed by the reduced capacity for dynamic exercise and the low ADP recovery rates after exercise [37,59].

#### 2.3.9. Increased Markers of Oxidative Stress

Higher levels of 4-hydroxynonenal-modified-proteins (4-HNE) as markers of oxidative stress have been observed in the quadriceps of 8-week-old McArdle mice compared to their WT counterparts [58]. Patients also show higher basal muscle levels of oxidative stress markers (4-HNE, 8-isoprostane and protein carbonyls) in skeletal muscle than healthy controls, together with a compensatory upregulation of the nuclear factor erythroid 2-related factor (Nrf2)-mediated antioxidant response [60,61].

#### 2.3.10. No Major Changes in the Autophagic Flux and Proteasome System

Higher protein levels of p62 and Beclin-1 have been reported in the quadriceps of 8-week-old McArdle mice with respect to WT mice; however, as no differences have been found for proteins of the autophagy–lysosomal system (phospho-unc-51-like kinase 1 (ULK1, Ser 555 and Ser 757), autophagy-related 16-like 1 (ATG16L) and lysosome-associated membrane glycoprotein 1 (LAMP1)) in McArdle mice along with the absence of massive accumulation of ubiquitaned proteins, it seems that the autophagic flux is not overall affected in the skeletal muscle of these animals [58]. Additionally, deregulation of the proteasome system has also been discarded as no significant differences in the protein levels of α and β catalytic subunits of the proteasome (αβ-P) exist between WT and McArdle mice [58].

#### 2.3.11. Alterations in CALCIUM Metabolism

Using two-dimensional electrophoresis, higher levels of the most acidic form of the (phosphorylated) of sarco (endo) plasmic reticulum ATPase 1 (SERCA-1) protein have been reported in the quadriceps of 8-week-old McArdle mice compared to their WT counterparts, suggesting an impairment in the catalytic cycle of this enzyme in the former [58]. In patients, indeed, total SERCA1 protein levels in skeletal muscle tissue are considerably lower than in healthy controls, with almost undetectable levels of the basic (unphosphorylated) form of this enzyme in two-dimensional electrophoresis [62].

#### 2.3.12. Disease Progression Differently Affects Distinct Muscles

The quadriceps and soleus muscles seem to be less histologically affected by disease progression than gastrocnemius, TA or EDL [48]. In the case of soleus muscle, this is supported by an increase in fiber size and centrally nucleated fibers with aging (i.e., 70 vs. 8-week-old mice), which is not found in the gastrocnemius, EDL, TA or quadriceps, suggesting that the fibers of the soleus might have a higher capacity to withstand repeated cycles of damage and regeneration. Furthermore, with aging the soleus muscle shows a trend to an increased reliance on glycolytic metabolism, as indicated by an increase in the percentage of glycolytic fibers paralleled by a reduction of oxidative fibers [47]. In the quadriceps muscle, low fibrosis accumulation and mild fiber size increases during aging, suggesting that this muscle is less affected by disease progression than the gastrocnemius, EDL or TA [48].

#### 2.3.13. Physiological and Molecular Adaptations to Endurance Exercise Training

When 8-week-old McArdle mice were subjected to an 8-week endurance exercise training program (forced treadmill running at submaximal intensities), a significant improvement in total running distance completed in an incremental test until exhaustion (an indicator of aerobic fitness) was observed compared to baseline (i.e., before training) [63]. However, although the relative improvement in aerobic fitness did not differ between McArdle and WT mice subjected to the same training protocol, the fitness levels of trained McArdle mice were still much lower (by 50%) than those of untrained WT mice. These results indicate that, similar to patients, McArdle mice adapt favorably to moderate-intensity endurance exercise training, but are unable to match the levels of healthy people whose ability to metabolize muscle glycogen is fully preserved. Furthermore, data provided by muscle proteome analyses revealed a remarkable difference between McArdle and WT mice in the protein networks involved in muscle tissue adaptations elicited by the exercise training intervention. Indeed, only three proteins showed a significant increased expression after training in both McArdle and WT mice: LIM and calponin homology domain-containing protein 1 (LIMCH1), poly (ADP-ribose) polymerase 1 (PARP-1) and tigger transposable element derived 4 (TIGD4) [63]. On the other hand, 74 and 123 proteins were differentially expressed with training in WT and McArdle mice, respectively, suggesting considerable differences in physiological adaptation to endurance exercise training between these two genotypes [63]. Particularly, mitogen-activated protein kinase 12 (MAPK12) protein expression was strongly induced in McArdle (but not in in WT) mice after the training period. MAPK12 is a serine-threonine kinase that acts as an essential component of the MAP kinase signal transduction pathway, plays an important role in myoblast differentiation and might also be involved in mitochondrial biogenesis and remodeling [63]. Pathway enrichment analysis of the highly and significantly expressed proteins in McArdle mice after endurance exercise training showed an enrichment in functions related to ATP synthesis and electron transport chain processes, β-oxidation of lipids, different steps in lipid and protein catabolism, cell death and regulation of necrotic processes. Conversely, the functions that were more represented in WT mice after endurance exercise training were those related with focal adhesions and cytoskeleton regulation, PI3K and mammalian target of rapamycin (mTOR) signaling pathways (the latter involved in the maintenance of skeletal muscle cell survival [63].

### 2.4. Zebrafish Model

A zebrafish model of McArdle disease has been recently reported [6,45] (Table 1). The zebrafish (*Danio rerio*) has two genes encoding GP-MM: phosphorylase, glycogen muscle, A (*Pygma*) and B (*Pygmb*) genes, respectively (with both sharing more than 80% of sequence identity with human *PYGM* gene). *Pygma* encodes a GP-MM protein of 842 amino acids, while *Pygmb* encodes three different GP-MM proteins of 842, 195 and 49 amino acids [45]. However, the two shorter variants lack important domains for glycogen phosphorylation such as the pyridoxal phosphate attachment site. Both *Pygma* and *Pygmb* are expressed in all analyzed developmental stages, although *Pygma* is clearly the dominant form in adult zebrafish. GP-MM protein was shown to increase its levels during zebrafish development, which correlated with a decrease in muscle glycogen levels. In parallel, GP-MM distribution in the muscles changed from dispersed to highly organized, showing a clearly striated pattern and co-localizing with α-actinin in the Z-line of sarcomeres [45]. These events corresponded to an increase in the energy demands, due to the first movements of the developing embryo. When *Pygma* and *Pygmb* genes were knocked down by injecting translation-blocking morpholino oligonucleotides, GP-MM protein levels were reduced in zebrafish morphants, which exhibited a curved body, reduced length, deformations in the tail region and pericardial edema. Additionally, they also showed decreased skeletal muscle formation, disintegrated muscle structure, loss of myofiber organization and accumulation of glycogen granules between sarcomeres and in the subsarcolemmal region, accompanied by a reduction of mobility and swimming speed [6,45].

## 3. Cell Cultures

Skeletal muscle cultures derived from human biopsies of McArdle disease patients do not constitute a good in vitro model of the disease, as they show GP activity [64,65], together with normal glycogen accumulation as determined by PAS staining [64]. Indeed, *PYGM* expression represents a small proportion of the total GP mRNA pool in human cell cultures either derived from affected or healthy individuals, whereas *PYGB* expression was predominant in myoblasts and *PYGB* and *PYGL* were both expressed in myotubes [66]. Skeletal muscle cultures derived from patients with McArdle disease do not show abnormal glycogen levels because *PYGB* and *PYGL* seem to be the predominant GP isoforms in this model [66]. By contrast, primary skeletal muscle cultures derived from McArdle mice are devoid of GP activity and accumulate large amounts of glycogen deposits, thereby representing a good in vitro model of McArdle disease [67]. Gene expression analysis of the different GP isoforms in these cultures show that while *Pygm* mRNA levels increase during skeletal muscle differentiation in WT cultures, both myoblasts and myotubes from McArdle mice express very low levels of *Pygm* mRNA [67]. On the other hand, no significant differences have been reported in myoblast or myotube *Pygb* mRNA levels between WT and McArdle mice, with *Pygl* mRNA not detected in either genotype [67]. At the protein level, GP-MM has only been found in WT myotubes, while neither GP-BB nor GP-LL have been detected by western blot analysis in myoblasts and myotubes from WT and McArdle mice [67].

## 4. Treatments

### 4.1. Upregulation Pygb/Pygl Expression in Mature Skeletal Muscle

In order to induce the expression of *Pygb* and/or *Pygl* genes in mature skeletal muscle that could compensate, at least partly, for the absence of GP-MM protein, two different compounds (notexin and valproate) have been evaluated in vitro and in vivo in these animal and cell culture models [46,68] (Table 3). Notexin is a myotoxic phospholipase derived from the venom of the Australian Tiger Snake (*Notechis scutatus scutatus*) [46]. This toxin causes hyalinization of muscle fibers with neutrophil infiltration soon after administration [69,70]. When it was administered to the McArdle sheep (*n* = 40 sheep aged 3 days to 3 years), as an intramuscular injection (*n* = 27) or as an application layered onto the surface of the muscle (*n* = 13), it led to a re-expression of *Pygb* and/or *Pygl* genes with a maximum peak at 10 days after administration [46]. A reduction of the glycogen levels in treated muscle fibers as well as an increase in strength of contraction was also observed [46]. This study reflects the potential functional benefit of stimulating the expression of these otherwise latent isoforms. In this regard, it has been described in the UCSC Genome Browser (http://genome-euro.ucsc.edu/index.html, accessed on 1 December 2021) that human, sheep and mouse *PYGB* and *PYGL* genes present CpG islands in their promoters, and, as such, their expression might be regulated through methylation. Furthermore, postnatal regulation of gene expression has been reported for many different CpG island containing genes [71]. Several studies have determined that valproic acid (VPA—a short chained fatty acid) can modulate the epigenome by inhibiting histone deacetylases and activating the expression of methylated genes through stimulation of active replication-independent demethylation [72]. When VPA was tested in vitro in WT and McArdle skeletal muscle cultures derived from the mouse model, an increase in *Pygb* mRNA and protein levels was observed in both cultures along with a significant reduction of the glycogen depots in McArdle myotubes [67]. VPA was also evaluated in vivo using the ovine model of McArdle disease [68]. In this study, three different approaches were evaluated: intramuscular injection of VPA, enteric administration of VPA solution and enteric administration of VPA tablets. In the first case, intramuscular injections of VPA (5 mL from a 0.5 g/30 mL solution) into the peroneus tertius and gluteobiceps muscles of the right pelvic limb and into the extensor carpi radialis and ulnaris lateralis of the right thoracic limb were performed in three 5-week-old affected lambs. Histologic analysis revealed significantly more GP-positive fibers in VPA-treated muscles than in control muscles injected with a saline solution, reaching the maximum peak of expression 9 days after injection. Of note, the appearance of GP-positive saline-treated fibers was probably caused by injection-related damage followed by muscle regeneration [68]. In the second approach, enteric administration of a VPA solution (20 to 60 mg/kg body weight per day for a maximum of 20 weeks) was performed bypassing the rumen, reticulum and omasum by administering VPA directly into the abomasum (using mushroom headed catheters) of eight lambs aged 12–18 days. Maximum VPA blood concentration was reached 2 h after administration, but declined 80% after 7 h. Blood analyses of VPA-treated animals showed an increase in CK and aspartate aminotransferase levels (consistent with diagnosis of McArdle disease), and a decrease in calcium concentration. GP positive fibers were observed in all the muscles analyzed with the exception of the diaphragm [68]. Finally, in the last approach, 6 affected ewes between 2.5 and 5 years of age were dosed with VPA in an enteric coated tablet form directly into the abomasum using a large Rumen cannula, while six untreated affected ewes of the same age were used as a control group [68]. The highest VPA blood concentration was found 1.5 h after dosing, and there was a ~60% decline after 7 h (almost 20% less than in the VPA solution administration). However, half of the treated ewes died using this method (at weeks 7 and 8 of a 16-week treatment period). One was found with bilateral multifocal acute bronchopneumonia with extensive pleuropericarditis and multifocal ulcerative abomasitis. Another presented necrotic enterocolitis and the third had abomasitis at the area adjacent to the catheter [68]. Blood analysis revealed increased CK and aspartate aminotransferase levels, while GP-positive fibers were found in all the muscles analyzed, with the exception of the triceps, supraspinatus and diaphragm. Physical activity measured as the time and distance walked (along a farm road in front of a quad bike driven at 5 km/h) before going into sternal recumbency was not significantly improved [68].

In light of the encouraging results obtained with VPA in the aforementioned preclinical studies, a phase II, open label, feasibility pilot trial was performed to assess the efficacy of VPA treatment in patients [76]. Sixteen patients with McArdle disease were treated for 6 months with VPA (20 mg/kg/day), and different endpoints were used to evaluate treatment efficacy: change in peak oxygen uptake (the gold standard measure of aerobic fitness), total distance walked on a 12-min walk test, forearm exercise test, histochemical expression of GP in skeletal muscles and “safety” blood parameters. However, no significant improvement in any of the clinical parameters was observed in treated patients. Histologically, there were few fibers that were positive for neonatal myosin but none showing GP staining, thereby suggesting that rather than regeneration these fibers presented an upregulation of neonatal myosin. Overall, this study showed that there was no clinically meaningful benefit in McArdle disease patients after 6 months of treatment with VPA [76].

### 4.2. Gene Therapy

Gene therapy could be a potentially useful therapy in McArdle disease, since there is preliminary evidence that a small amount of enzyme is enough to ameliorate the disease symptoms [78]. Gene therapy was tested in vivo for the first time in the ovine model (Table 3). Treatment was performed in sheep aged between 2 days and 14 months via semitendinosus intramuscular injections of adenovirus 5 (AdV5) and adeno-associated virus serotype 2 (AAV2), both containing the LacZ reporter (control) or human *PYGM* cDNA. GP expression was observed in the site of injection and the protein was functional, as indicated by the correlation with GP-MM positive fibers and a reduction of glycogen accumulation [74]. GP-MM was observed in animals injected with *PYGM*, whereas non-muscular isoforms were re-expressed in animals injected with LacZ reporter as a consequence of inflammation and regeneration. Despite the good results, expression of *PYGM* appeared to decrease with time, possibly due to an immune response against the human *PYGM* and to the immune reactions of the specific viral vectors [74]. However, important advances have been made since year 2008 in the usage and design of viral vectors. In fact, different trials using AAV6/8/9 have recently shown that these vectors efficiently transduce skeletal muscle and exhibit low immunogenicity, in fact providing good results in large animal models of Duchenne muscular dystrophy and myotubular myopathy [79,80,81,82]. Hence, systemic delivery of rAAV2/8 expressing murine *Pygm* under the control of a synthetic triple muscle-specific CK (MCK) gene promoter. MCK gene promoter was evaluated in McArdle mice at post-natal days 1–3 [75] (Table 3). Detectable levels (0.5 to 21% of WT levels) of *Pygm* were observed in the quadriceps of treated mice 8 weeks post-injection. In addition, a significant reduction was reported in muscle glycogen levels together with an improved performance in voluntary wheel running [75]. However, no improvement was found in treated mice for performance in the hang wire test, probably reflecting that the level of *Pygm* expression achieved was enough to meet the energy requirements of sub-maximal endurance exercise, but not to support short bursts of maximal effort [75]. Further studies to achieve a higher expression of the transgene along with a more complete correction of the disease phenotype should be performed before this approach can be proposed for patients.

### 4.3. Read-Through

Because the most prevalent pathogenic variants in patients with McArdle disease are premature termination codons (PTC), read-through agents (RTA), which are able to induce the ribosome to bypass a PTC, appear as good treatment candidates for this condition [83,84] (Table 3).

RTA were first evaluated in McArdle disease in a preliminary trial with a short-term treatment using gentamicin in patients with a PTC, which failed to normalize 31P-magnetic resonance spectroscopy of GP-MM deficiency in the skeletal muscle [77]. Seven years later, RTA were again evaluated as potential therapeutic compounds for McArdle disease, and this time read-through activity was observed in non-muscle cell cultures transiently transfected with p.R50X-green fluorescent protein (GFP) constructs when treated with an aminoglycoside antibiotic, G418 [85]. Recently, in order to further clarify the potential benefit of these compounds as therapeutic agents, a wider battery of RTAs (including amlexanox, Ataluren, RTC13, RTC14 and G418, among others) were tested again in transiently transfected cells with p.R50*-GFP but also in cells stably expressing these constructs and in skeletal muscle cultures derived from the McArdle mouse model [73]. In this study, no read-through induction was observed in any of the cells with the different RTA tested. In addition, as read-through efficiency is influenced by the stop codon composition and its surrounding sequence, the p.R50* *Pygm* context sequence was analyzed and compared with 30 different PTC sequences (including 15 nucleotides upstream and downstream from the PTC) with reported positive read-through and 29 PTC sequences with reported negative read-through induction. Compared with these analyzed sequences, the *Pygm* sequence of McArdle mice showed the TGA stop codon as well as a G nucleotide at position −9, and a C at position −3 (being the first nucleotide of the PTC where the position was defined as +1), which are commonly found in the PTC sequences with positive read-through induction, but also presented a C at −2, more frequently found in PTC sequences with negative read-through induction [73]. In addition, the *Pygm* sequence did show a C at +4, which has been described to promote PTC read-through [86]. Overall, the results from these three independent studies failed to provide evidence that RTA might be useful therapeutic agents for McArdle disease patients. However, the list of new potential RTA increases every day and the read-through capacity of compounds, such as NB74, NB84, NB124, GJ071, GJ072, BZ6, BZ16 and clitozine, among others, should be also tested in McArdle disease in in vitro and in vivo models.

## 5. Critical Discussion

Owing to the difficulty of performing mechanistic studies in patients that often rely on invasive techniques, preclinical models have been used for decades, thereby contributing to gain insight into the pathophysiology and pathobiology of human diseases [71]. In this context, at least 42 different animal models (including 15 naturally occurring animals and 26 genetically-modified mouse models) have been reported for the different GSD [33], and specifically four different animal models for McArdle disease (bovine, ovine, mouse and zebrafish models). The bovine and ovine naturally occurring models were the first to be described (in 1995 and 1997, respectively) [41,43]. These two models helped to gain insight into the pathophysiological mechanisms of the disease [41,42,43,46,68,74], and allowed the initiation of the first preclinical trials using in vivo models [46,68,74]. Yet the development of the McArdle mouse model added an additional burst in the histological, molecular, biochemical and physiological characterization of the disease, principally based on the fact that animal manipulation and maintenance, as well as sharing between different research groups, is easier when using mice than bigger animals. The mouse model, as in the case of the bovine and ovine models, faithfully reproduces the disease phenotype found in patients. However, the huge glycogen depots in the skeletal muscle of McArdle mice (20 to 80 times higher than WT mice [40]) in comparison to patients (2–3 times higher than healthy people [87]) with subsequent differences in muscle tissue structural alterations represents a major difference between the phenotype of mice and patients. This limitation is also found in other GSD mouse models (e.g., GSD II, III and IV, with >20 times higher glycogen levels than WT mice) [49] and might be attributable to the faster metabolic rates of these animals, leading to an accelerated accumulation of glycogen in affected tissues. By contrast, in the McArdle bovine and ovine models, muscle glycogen concentrations are 1.6 and 2.2 times higher, respectively, than in control animals [46,88]. In this regard, large animals such as cows and sheep represent better models to reproduce disease phenotypes than small models, as they show similarities with regard to humans in organ and body size, muscle bulk, and overall physio-pathological and metabolic characteristics [89]. In particular, the ovine model presents a body and muscle mass throughout life that is similar to humans [74]. However, there are intrinsic difficulties in working with cows and sheep compared to mouse models, such as manipulation, phenotype characterization (e.g., exercise testing), breeding, housing costs, space availability and the possibility of sharing animals between different research groups (Table 1) [49].

Another major difference in the disease phenotype between patients and the mouse model is the high frequency of premature death in McArdle mice (~85% of the animals that are born), mainly occurring in the perinatal and post-weaning periods [48,49]. As with the highly elevated glycogen depots, perinatal and post-weaning death has also been reported in different GSD mouse models (GSD 0b, Ia, Ib, IV, VII and XV) [48,49]. A possible explanation for this phenomenon might be related to the deficit of glycogen-driven glucose availability in affected tissues to meet basic energy requirements, since glycogen might play a crucial role in energy supply during the neonatal period due to the low content of glucose in milk [90]. However, perinatal death has not been reported in the bovine, ovine or zebrafish models of McArdle disease.

Despite the aforementioned phenotype differences between McArdle mice and patients, the mouse model is allowing to characterize physio-pathological mechanisms of McArdle disease for the first time, such as specific fiber type degeneration, calcium and mitochondrial metabolism, glycogen and glucose turnover signaling pathways or autophagy processes.

The zebrafish model of the disease offers some advantages with respect the bovine, ovine and mouse models, such as production of a large number of embryos, short life cycle (at least vs. the bovine and ovine model, with maturity reached within 3 months) and less expensive maintenance [6,45] (Table 1). In addition, the glycogen concentrations reported for this model (1.3 times higher than in controls) are relatively similar to the actual findings in humans, as opposed to the mouse model. However, there are also some disadvantages associated to this model, mainly related to the fact that zebrafish is not a mammal, which might hamper its use in preclinical drug trials [6].

The different in vitro and in vivo models of McArdle disease have allowed to assess potential molecular treatments at the preclinical stage (Table 3). From these, only the VPA-induced upregulation of the *PYGB/PYGL* isoforms in mature skeletal muscle has further been tested in humans in a pilot clinical trial. Unfortunately, no clinical improvement was reported in VPA-treated patients [76], suggesting that it does not seem worthwhile to pursue further studies with this drug. Nonetheless, other compounds with epigenetic regulation capacity might be useful for the treatment of McArdle disease. With regard to gene therapy, although encouraging, positive results have been reported with the ovine and mouse model [74,75], more studies are still needed in order to induce actual increases in the expression of the transgene in the skeletal muscle to further reverse the clinical phenotype before this approach can be proposed for patients. Finally, RTA agents have also been evaluated in vitro both in transiently transfected cells and in skeletal muscle cultures derived from the McArdle mouse model, and the absence of read-through induction with the different tested compounds [73,91], has prevented further studies using in vivo models. This being said, because the number of potential RTA is constantly increasing, it cannot be completely ruled out that a new RTA might be useful for the treatment of McArdle disease.

In conclusion, the different animal models of McArdle disease, despite presenting some differences with the typical patient’s phenotype, might allow for a deep study of the different features of the disease while representing a necessary preclinical step to assessed the efficacy and safety of possible treatments before they are tested in patients.

## Figures and Tables

**Table 1:** The different preclinical research in vivo models for McArdle disease. Abbreviations: AST: Aspartate aminotransferase; CK: Creatine kinase; f.i: fold increase; N.A: not applicable; ↑: increase; ↓: decrease.

	Bovine Model	Ovine Model	Mouse Model	Zebrafish Model
Origin	Davis, CA, USA	Western Australia	Barcelona, Spain	Wroclaw, Poland
First Report	1995 [41]	1997 [43]	2012 [44]	2020 [45]
Type of model	Spontaneous	Spontaneous	Knock-in	Knock down (morpholino oligonucleotides)
Mutation	c.1468C>T (p.R490W)	c.2380-1G>A	c.148A>T (p.R50*)	N.A
Exercise intolerance	Yes	Yes	Yes	Yes
Large glycogen depots	Yes (~1.6 f.i)	Yes (~2.2 f.i)	Yes (~20–80 f.i)	Yes (~1.3 f.i)
Blood analyses	K^+^↑, Phosphate ↓, Ca^2+^↑, CK↑	CK↑, AST↑, Ca^2+^↓	CK↑, NH^3+^↑, Glucose↓, Lactate↓	N.A
Advantages	1.Muscle fiber type compostion similar to humans. 2.Mitochondria density volume per fiber volume (2–5%) similar to humans	1.Animal size and muscle mass comparable to humans. 2. Muscle fiber type composition similar to humans. 3. Mitochondria density volume per fiber volume (2–5%) similar to humans	1. Presents a complete McArdle disease phenotype. 2.Presents the most common mutation in Caucasian patients (p.R50X). 3. Easy to manipulate. 4. Easy to share with other reseach groups 5. Low mantainenance costs	1. Phenotype similar to McArdle disease patients. 2. Low maintenace costs. 3. Easy to obtain large number of animals for experimentation (100–200 eggs per week)
Disadvantages	1.Difficult to obtain animals for experimentation: long gestation period (270–295 days) + 1 calf per birth. 2. Difficult to manipulate (average cow weight: 450 kg). 3. Difficult to share with other research groups. 4. High maintenance costs.	1.Difficult to obtain animals for experimentation: long gestation period (147 days) + 1–3 lambs per birth. 2. Difficult to manipulate (sheep weight: 40–100 kg). 3. Difficult to share with other research groups. 4. High maintenance costs.	1.High perinatal mortality. 2.Higher glycogen accumulation in comparison to patients. 3. Different fiber type composition (predominantly type IIX and IIB fibers) in comparison to humans (predominantly type I and IIA fibers). 4. Different mitochondria density volume per fiber volume (30%) in comparison to humans (2–5%)	1. Is not a mammal. 2. Poor water solubility of some chemicals for drug testing. 3. Doses for toxicological tests are very different from mammals.

**Table 2:** Changes in the relative protein levels of McArdle mice compared to WT mice. Horizontal double black arrows indicate no differences in the protein levels between WT and McArdle mice. Vertical green arrows (↑) indicate upregulation in McArdle mice, while vertical red arrows (↓) indicate downregulation. Horizontal arrows (↔) indicate no significant changes. Abbreviations: n.d: not determined; wo: weeks old; yo: years old.

		McArdle Mice											McArdle Patients
		Soleus		Gastrocnemius			EDL		TA			Quadriceps	Vastus Lateralis
		8 wo	20 wo	8 wo	35 wo	70 wo	8 wo	20 wo	8 wo	20 wo	70 wo	20 wo	Avg = 38.4 yo (rg = 18–62)
Relative protein levels vs. WT	GDE	↑	↔	↔	n.d	n.d	↔	↔	n.d	↔	n.d	↔	↔
	GS	↓	↔	↓	↔	↔	↓	↔	↔	↔	↑	↑	↑
	pGS	↑	↔	↑	↑	↑	↑	↔	n.d	↑	↑	↑	↑
	GBE	↔	↔	↑	n.d	n.d	↑	↔	n.d	↔	n.d	↑	↑
	pAMPK	n.d	↔	n.d	n.d	n.d	n.d	↔	n.d	↑	n.d	↑	↔
	pAKT	n.d	↔	n.d	n.d	n.d	n.d	↓	n.d	↔	n.d	↔	↔
	pGSK3	n.d	↔	n.d	n.d	n.d	n.d	↔	n.d	↔	n.d	↔	↔
	pTBC1D1	n.d	↔	n.d	n.d	n.d	n.d	↔	n.d	↓	n.d	↔	↔
	pTBC1D4	n.d	↔	n.d	n.d	n.d	n.d	↔	n.d	↔	n.d	↑	↔
	Glut4	n.d	↔	n.d	n.d	n.d	n.d	↔	n.d	↔	n.d	↑	↑
	Hexokinase II	n.d	↔	n.d	n.d	n.d	n.d	↔	n.d	↔	n.d	↔	↑
	F6PK	n.d	↔	n.d	n.d	n.d	n.d	↔	n.d	↔	n.d	↔	↑
	pCaMKII	n.d	↔	n.d	n.d	n.d	n.d	↔	n.d	↔	n.d	↓	↑
	pPKA	n.d	↔	n.d	n.d	n.d	n.d	↔	n.d	↔	n.d	↔	↑
	PHKA	n.d	↔	n.d	n.d	n.d	n.d	↔	n.d	↔	n.d	↑	↑

**Table 3:** List of therapeutic approaches tested in the different preclinical research models, and if further tested in humans, data are also shown.

	Pygb/Pygl Upregulation		Gene Therapy		Read-Through
	Notexin	VPA	AdV5/AAV2	rAAV2/8	RTAs
**In vitro**					
Mouse myotubes	Not tested	Dose dependent increase of Pygb expression. Dose dependent reduction of glycogen levels [67].	Not tested	Not tested	No p.R50X Pygm read-through was observed [73].
**In vivo**					
Bovine model	Not tested	Not tested	Not tested	Not tested	Not tested
Ovine model	Re-expression of Pygb/Pygl. Reduction in glycogen content. Increase in contraction strenght [46].	Presence of GP positive fibers. No significant improvement in exercise capacity [68].	Presence of GP positive fibers. Decrease in glycogen content. PYGM expression decreased with time [74].	Not tested	Not tested
Mouse model	Not tested	Not tested	Not tested	Re-expressión of Pygm (0.5–21% of control values). Decreased glycogen levels. Increased voluntary wheel running. Lack of improvement in hang wire exercise capacity [75].	Not tested
Zebrafish model	Not tested	Not tested	Not tested	Not tested	Not tested
Human patients	Not tested	No significant clinical benefits were observed [76].	Not tested	Not tested	No p.R50X Pygm read-through was observed [77].

## Data Availability

Not applicable.

## References

[1] McArdle B. (1951). Myopathy due to a defect in muscle glycogen breakdown. Clin. Sci..

[2] Lucia A., Ruiz J.R., Santalla A., Nogales-Gadea G., Rubio J.C., Garcia-Consuegra I., Cabello A., Pérez M., Teijeira S., Vieitez I. (2012). Genotypic and phenotypic features of McArdle disease: Insights from the Spanish national registry. J. Neurol. Neurosurg. Psychiatry.

[3] Vissing J., Haller R.G. (2003). The effect of oral sucrose on exercise tolerance in patients with McArdle’s disease. N. Engl. J. Med..

[4] Nogales-Gadea G., Santalla A., Brull A., de Luna N., Lucia A., Pinos T. (2015). The pathogenomics of McArdle disease--genes, enzymes, models, and therapeutic implications. J. Inherit. Metab. Dis..

[5] Uhlén M., Fagerberg L., Hallström B.M., Lindskog C., Oksvold P., Mardinoglu A., Sivertsson Å., Kampf C., Sjöstedt E., Asplund A. (2015). Proteomics. Tissue-Based Map of the Human Proteome. Science.

[6] Migocka-Patrzalek M., Elias M. (2021). Muscle Glycogen Phosphorylase and Its Functional Partners in Health and Disease. Cells.

[7] Schmid H., Dolderer B., Thiess U., Verleysdonk S., Hamprecht B. (2008). Renal expression of the brain and muscle isoforms of glycogen phosphorylase in different cell types. Neurochem. Res..

[8] Abugessaisa I., Noguchi S., Hasegawa A., Harshbarger J., Kondo A., Lizio M., Severin J., Carninci P., Kawaji H., Kasukawa T. (2017). FANTOM5 CAGE profiles of human and mouse reprocessed for GRCh38 and GRCm38 genome assemblies. Sci. Data.

[9] Llavero F., Montoro M.L., Sastre A.A., Fernández-Moreno D., Lacerda H.M., Parada L.A., Lucia A., Zugaza J.L. (2019). Epidermal growth factor receptor controls glycogen phosphorylase in T cells through small GTPases of the RAS family. J. Biol. Chem..

[10] Tsujino S., Shanske S., Nonaka I., Di Mauro S. (1995). The molecular genetic basis of myophosphorylase deficiency (McArdle’s disease). Muscle Nerve Suppl..

[11] Nogales-Gadea G., Brull A., Santalla A., Andreu A.L., Arenas J., Martin M.A., Lucia A., de Luna N., Pinós T. (2015). McArdle Disease: Update of Reported Mutations and Polymorphisms in the PYGM Gene. Hum. Mutat..

[12] Petrou P., Pantzaris M., Dionysiou M., Drousiotou A., Kyriakides T. (2015). Minimally symptomatic mcardle disease, expanding the genotype-phenotype spectrum. Muscle Nerve.

[13] Garcia-Consuegra I., Blázquez A., Rubio J.C., Arenas J., Ballester-Lopez A., González-Quintana A., Andreu A.L., Pinós T., Coll-Cantí J., Lucia A. (2016). Taking advantage of an old concept, “illegitimate transcription”, for a proposed novel method of genetic diagnosis of McArdle disease. Genet. Med..

[14] Cheraud C., Froissart R., Lannes B., Echaniz-Laguna A. (2018). Novel variant in the PYGM gene causing late-onset limb-girdle myopathy, ptosis, and camptocormia. Muscle Nerve.

[15] Inal-Gültekin G., Toptaş-Hekimoğlu B., Görmez Z., Gelişin Ö., Durmuş H., Ergüner B., Demirci H., Sağıroğlu M.Ş., Parman Y., Deymeer F. (2017). Myophosphorylase (PYGM) mutations determined by next generation sequencing in a cohort from Turkey with McArdle disease. Neuromuscul. Disord..

[16] Santalla A., Nogales-Gadea G., Encinar A.B., Vieitez I., González-Quintana A., Serrano-Lorenzo P., Consuegra I.G., Asensio S., Ballester-Lopez A., Pintos-Morell G. (2017). Genotypic and phenotypic features of all Spanish patients with McArdle disease: A 2016 update. BMC Genom..

[17] Nabavi Nouri M., Lamhonwah A.M., Tein I. (2018). Novel myophosphorylase mutation (p.Arg94Pro) with progressive exercise intolerance. Clin. Case Rep..

[18] Mahroo O.A., Khan K.N., Wright G., Ockrim Z., Scalco R.S., Robson A.G., Tufail A., Michaelides M., Quinlivan R., Webster A.R. (2019). Retinopathy Associated with Biallelic Mutations in PYGM (McArdle Disease). Ophthalmology.

[19] Lorenzoni P.J., Werneck L.C., Kay C.S.K., Arndt R.C., Silvado C.E.S., Scola R.H. (2020). Single-centre experience on genotypic and phenotypic features of southern Brazilian patients with McArdle disease. Acta Neurol. Belg..

[20] Satoh A., Hirashio S., Arima T., Yamada Y., Irifuku T., Ishibashi H., Motoda A., Sueda Y., Masaki T. (2019). Novel Asp511Thr mutation in McArdle disease with acute kidney injury caused by rhabdomyolysis. CEN Case Rep..

[21] Gomes C.P., da Silva A.M.S., Zanoteli E., Pesquero J.B. (2020). A new mutation in PYGM causing McArdle disease in a Brazilian patient. Acta Neurol. Belg..

[22] Xie R.R., Yang Y.B., Jin P. (2020). Identification of a novel PYGM mutation in a McArdle disease patient misdiagnosed as hypokalemic periodic paralysis. J. Endocrinol. Investig..

[23] Chocair P.R., Mohrbacher S., Neves P.D.M.D.M., Pereira L.V.B., Oliveira E.S., Nardotto L.L., Bales A.M., Sato V.A.H., Silva S.N., Ferreira B.M.C. (2020). An elderly diabetic patient with McArdle disease and recurrent rhabdomyolysis: A potential association with late hypoinsulinemia?. BMC Geriatr..

[24] Kang J.H., Park J.H., Park J.S., Lee S.K., Lee S., Baik H.W. (2021). Molecular diagnosis of McArdle disease using whole-exome sequencing. Exp. Ther. Med..

[25] Pizzamiglio C., Mahroo O.A., Khan K.N., Patasin M., Quinlivan R. (2021). Phenotype and genotype of 197 British patients with McArdle disease: An observational single-centre study. J. Inherit. Metab. Dis..

[26] Quinlivan R., Buckley J., James M., Twist A., Ball S., Duno M., Vissing J., Bruno C., Cassandrini D., Roberts M. (2010). McArdle disease: A clinical review. J. Neurol. Neurosurg. Psychiatry.

[27] Bruno C., Cassandrini D., Martinuzzi A., Toscano A., Moggio M., Morandi L., Servidei S., Mongini T., Angelini C., Musumeci O. (2006). McArdle disease: The mutation spectrum of PYGM in a large Italian cohort. Hum. Mutat..

[28] Bartram C., Edwards R.H., Clague J., Beynon R.J. (1993). McArdle’s disease: A nonsense mutation in exon 1 of the muscle glycogen phosphorylase gene explains some but not all cases. Hum. Mol. Genet..

[29] El-Schahawi M., Tsujino S., Shanske S., Di Mauro S. (1996). Diagnosis of McArdle’s disease by molecular genetic analysis of blood. Neurology.

[30] Martín M.Á., Rubio J.C., Wevers R., Van Engelen B.G.M., Steenbergen G.C.H., Van Diggelen O.P., De Visser M., De Die-Smulders C., Blazquez A., Andreu A.L. (2004). Molecular Analysis of Myophosphorylase Deficiency in Dutch Patients with McArdle’s Disease. Ann. Hum. Genet..

[31] Aquaron R., Berge-Lefranc J.L., Pellissier J.F., Montfort M.F., Mayan M., Figarella-Branger D., Coquet M., Serratrice G., Pouget J. (2007). Molecular characterization of myophosphorylase deficiency (McArdle disease) in 34 patients from Southern France: Identification of 10 new mutations. Absence of genotype-phenotype correlation. Neuromuscul. Disord. NMD.

[32] Gurgel-Giannetti J., Nogales-Gadea G., van der Linden H., Bellard T.M., Brasileiro Filho G., Giannetti A.V., de Castro Concentino E.L., Vainzof M. (2013). Clinical and molecular characterization of McArdle’s disease in Brazilian patients. Neuromol. Med..

[33] Deschauer M., Morgenroth A., Joshi P.R., Glaser D., Chinnery P.F., Aasly J., Schreiber H., Knape M., Zierz S., Vorgerd M. (2007). Analysis of spectrum and frequencies of mutations in McArdle disease. J. Neurol..

[34] Martin M.A., Rubio J.C., Buchbinder J., Fernandez-Hojas R., del Hoyo P., Teijeira S., Gámez J., Navarro C., Fernández J.M., Cabello A. (2001). Molecular heterogeneity of myophosphorylase deficiency (McArdle’s disease): A genotype-phenotype correlation study. Ann. Neurol..

[35] Martinuzzi A., Sartori E., Fanin M., Nascimbeni A., Valente L., Angelini C., Siciliano G., Mongini T., Tonin P., Tomelleri G. (2003). Phenotype modulators in myophosphorylase deficiency. Ann. Neurol..

[36] Paradas C., Fernandez-Cadenas I., Gallardo E., Llige D., Arenas J., Illa I., Andreu A.L. (2005). Variable presentation of the clinical phenotype of McArdle’s disease in a kindred harbouring a novel compound genotype in the muscle glycogen phosphorylase gene. Neurosci. Lett..

[37] Lucia A., Nogales-Gadea G., Perez M., Martin M.A., Andreu A.L., Arenas J. (2008). McArdle disease: What do neurologists need to know?. Nat. Clin. Pract. Neurol..

[38] Preisler N., Cohen J., Vissing C.R., Madsen K.L., Heinicke K., Sharp L.J., Phillips L., Romain N., Park S.Y., Newby M. (2017). Impaired glycogen breakdown and synthesis in phosphoglucomutase 1 deficiency. Mol. Genet. Metab..

[39] Santalla A., Nogales-Gadea G., Ortenblad N., Brull A., de Luna N., Pinos T., Phillips L., Romain N., Park S.Y., Newby M. (2014). McArdle disease: A unique study model in sports medicine. Sports Med..

[40] Brull A., de Luna N., Blanco-Grau A., Lucia A., Martin M.A., Arenas J., Lucia A. (2015). Phenotype consequences of myophosphorylase dysfunction: Insights from the McArdle mouse model. J. Physiol..

[41] Angelos S., Valberg S.J., Smith B.P., McQuarrie P.S., Shanske S., Tsujino S., DiMauro S., Cardinet G.H. (1995). Myophosphorylase deficiency associated with rhabdomyolysis and exercise intolerance in 6 related charolais cattle. Muscle Nerve.

[42] Tsujino S., Shanske S., Valberg S.J., Cardinet G.H., Smith B.P., Di Mauro S. (1996). Cloning of bovine muscle glycogen phosphorylase cDNA and identification of a mutation in cattle with myophosphorylase deficiency, an animal model for McArdle’s disease. Neuromuscul. Disord. NMD.

[43] Tan P., Allen J.G., Wilton S.D., Akkari P.A., Huxtable C.R., Laing N.G. (1997). A splice-site mutation causing ovine McArdle’s disease. Neuromuscul. Disord. NMD.

[44] Nogales-Gadea G., Pinós T., Lucia A., Arenas J., Cámara Y., Brull A., De Luna N., Martín M.A., Garcia-Arumí E., Marti R. (2012). Knock-in mice for the R50X mutation in the PYGM gene present with McArdle disease. Brain.

[45] Migocka-Patrzalek M., Lewicka A., Elias M., Daczewska M. (2020). The effect of muscle glycogen phosphorylase (Pygm) knockdown on zebrafish morphology. Int. J. Biochem. Cell Biol..

[46] Howell J.M., Walker K.R., Creed K.E., Dunton E., Davies L., Quinlivan R., Karpati G. (2014). Phosphorylase re-expression, increase in the force of contraction and decreased fatigue following notexin-induced muscle damage and regeneration in the ovine model of McArdle disease. Neuromuscul. Disord. NMD.

[47] Krag T.O., Pinos T., Nielsen T.L., Brull A., Andreu A.L., Vissing J. (2016). Differential Muscle Involvement in Mice and Humans Affected by McArdle Disease. J. Neuropathol. Exp. Neurol..

[48] Real-Martinez A., Brull A., Huerta J., Tarrasó G., Lucia A., Martin M., Arenas J., Andreu A.L., Nogales-Gadea G., Vissing J. (2019). Low survival rate and muscle fiber-dependent aging effects in the McArdle disease mouse model. Sci. Rep..

[49] Almodovar-Paya A., Villarreal-Salazar M., de Luna N., Nogales-Gadea G., Real-Martinez A., Andreu A.L., Martín M.A., Arenas J., Lucia A., Vissing J. (2020). Preclinical Research in Glycogen Storage Diseases: A Comprehensive Review of Current Animal Models. Int. J. Mol. Sci..

[50] Nielsen J.N., Wojtaszewski J.F.P., Haller R.G., Hardie D.G., Kemp B.E., Richter E.A., Vissing J. (2002). Role of 5′AMP-activated protein kinase in glycogen synthase activity and glucose utilization: Insights from patients with McArdle’s disease. J. Physiol..

[51] Mineo I., Kono N., Shimizu T., Hara N., Yamada Y., Sumi S., Nonaka K., Tarui S. (1985). Excess purine degradation in exercising muscles of patients with glycogen storage disease types V and VII. J. Clin. Investig..

[52] Brooke M.H., Patterson V.H., Kaiser K.K. (1983). Hypoxanthine and Mcardle disease: A clue to metabolic stress in the working forearm. Muscle Nerve.

[53] Rubio J.C., Pérez M., Maté-Muñoz J.L., García-Consuegra I., Chamorro-Viña C., Del Valle M.F., Andreu A.L., Martin M.A., Arenas J., Lucia A. (2007). AMPD1 Genotypes and Exercise Capacity in McArdle Patients. Int. J. Sports Med..

[54] Nielsen T.L., Pinós T., Brull A., Vissing J., Krag T.O. (2018). Exercising with blocked muscle glycogenolysis: Adaptation in the McArdle mouse. Mol. Genet. Metab..

[55] Kohn T.A., Noakes T.D., Rae D.E., Rubio J.C., Santalla A., Nogales-Gadea G., Pinós T., Martín M.A., Arenas J., Lucia A. (2014). McArdle disease does not affect skeletal muscle fibre type profiles in humans. Biol. Open.

[56] Murphy R.M., Xu H., Latchman H., Larkins N.T., Gooley P.R., Stapleton D.I. (2012). Single fiber analyses of glycogen-related proteins reveal their differential association with glycogen in rat skeletal muscle. Am. J. Physiol. Cell Physiol..

[57] Krag T.O., Pinós T., Nielsen T.L., Duran J., Garcia-Rocha M., Andreu A.L., Vissing J. (2016). Differential glucose metabolism in mice and humans affected by McArdle disease. Am. J. Physiol. Integr. Comp. Physiol..

[58] Fiuza-Luces C., Nogales-Gadea G., Garcia-Consuegra I., Pareja-Galeano H., Rufian-Vazquez L., Perez L.M., Andreu A.L., Arenas J., Martín M.A., Pinós T. (2016). Muscle Signaling in Exercise Intolerance: Insights from the McArdle Mouse Model. Med. Sci. Sports Exerc..

[59] De Stefano N., Argov Z., Matthews P.M., Karpati G., Arnold D.L. (1996). Impairment of muscle mitochondrial oxidative metabolism in McArdles’s disease. Muscle Nerve.

[60] Kitaoka Y., Ogborn D.I., Nilsson M.I., Mocellin N.J., MacNeil L.G., Tarnopolsky M.A. (2013). Oxidative stress and Nrf2 signaling in McArdle disease. Mol. Genet. Metab..

[61] Kaczor J.J., Robertshaw H.A., Tarnopolsky M.A. (2017). Higher oxidative stress in skeletal muscle of McArdle disease patients. Mol. Genet. Metab. Rep..

[62] Nogales-Gadea G., Consuegra-García I., Rubio J.C., Arenas J., Cuadros M., Cámara Y., Torres-Torronteras J., Fiuza-Luces C., Lucia A., Martín M.A. (2012). A Transcriptomic Approach to Search for Novel Phenotypic Regulators in McArdle Disease. PLoS ONE.

[63] Fiuza-Luces C., Santos-Lozano A., Llavero F., Campo R., Nogales-Gadea G., Díez-Bermejo J., Baladrón C., González-Murillo Á., Arenas J., Martín M.A. (2018). Muscle molecular adaptations to endurance exercise training are conditioned by glycogen availability: A proteomics-based analysis in the McArdle mouse model. J. Physiol..

[64] Martinuzzi A., Vergani L., Carrozzo R., Fanin M., Bartoloni L., Angelini C., Engel W.K. (1993). Expression of muscle-type phosphorylase in innervated and aneural cultured muscle of patients with myophosphorylase deficiency. J. Clin. Investig..

[65] Meienhofer M.C., Askanas V., Proux-Daegelen D., Dreyfus J.C., Engel W.K. (1977). Muscle-type phosphorylase activity present in muscle cells cultured from three patients with myophosphorylase deficiency. Arch. Neurol..

[66] Nogales-Gadea G., Mormeneo E., Garcia-Consuegra I., Rubio J.C., Orozco A., Arenas J., Martín M.A., Lucia A., Gomez-Foix A.M., Marti R. (2010). Expression of glycogen phosphorylase isoforms in cultured muscle from patients with McArdle’s disease carrying the p.R771PfsX33 PYGM mutation. PLoS ONE.

[67] de Luna N., Brull A., Guiu J.M., Lucia A., Martín M.Á., Arenas J., Martí R., Andreu A.L., Pinós T. (2015). Sodium valproate increases the brain isoform of glycogen phosphorylase: Looking for a compensation mechanism in McArdle disease using a mouse primary skeletal-muscle culture in vitro. Dis. Model. Mech..

[68] Howell J.M., Dunton E., Creed K.E., Quinlivan R., Sewry C. (2015). Investigating sodium valproate as a treatment for McArdle disease in sheep. Neuromuscul. Disord. NMD.

[69] Harris J.B., Johnson M.A. (1978). Further observations on the pathological responses of rat skeletal muscle to toxins isolated from the venom of the Australian tiger snake, Notechis scutatus scutatus. Clin. Exp. Pharmacol. Physiol..

[70] Sharp N.J., Kornegay J.N., Bartlett R.J., Hung W.Y., Dykstra M.J. (1993). Notexin-induced muscle injury in the dog. J. Neurol. Sci..

[71] Numata S., Ye T., Hyde T.M., Guitart-Navarro X., Tao R., Wininger M., Colantuoni C., Weinberger D.R., Kleinman J.E., Lipska B.K. (2012). DNA methylation signatures in development and aging of the human prefrontal cortex. Am. J. Hum. Genet..

[72] Detich N., Bovenzi V., Szyf M. (2003). Valproate induces replication-independent active DNA demethylation. J. Biol. Chem..

[73] Tarrasó G., Real-Martinez A., Parés M., Romero-Cortadellas L., Puigros L., Moya L., de Luna N., Brull A., Martín M.A., Arenas J. (2020). Absence of p.R50X Pygm read-through in McArdle disease cellular models. Dis. Model. Mech..

[74] Howell J.M., Walker K.R., Davies L., Dunton E., Everaardt A., Laing N., Karpati G. (2008). Adenovirus and adeno-associated virus-mediated delivery of human myophosphorylase cDNA and LacZ cDNA to muscle in the ovine model of McArdle’s disease: Expression and re-expression of glycogen phosphorylase. Neuromuscul. Disord. NMD.

[75] McNamara E.L., Taylor R.L., Clayton J., Goullee H., Dilworth K.L., Pinós T., Brull A., Alexander I.E., Lisowski L., Ravenscroft G. (2019). Systemic AAV8-mediated delivery of a functional copy of muscle glycogen phosphorylase (Pygm) ameliorates disease in a murine model of McArdle disease. Hum. Mol. Genet..

[76] Scalco R.S., Stemmerik M., Løkken N., Vissing C.R., Madsen K.L., Michalak Z., Pattni J., Godfrey R., Samandouras G., Bassett P. (2020). Results of an open label feasibility study of sodium valproate in people with McArdle disease. Neuromuscul. Disord..

[77] Schroers A., Kley R.A., Stachon A., Horvath R., Lochmuller H., Zange J., Vorgerd M. (2006). Gentamicin treatment in McArdle disease: Failure to correct myophosphorylase deficiency. Neurology.

[78] Vissing J., Duno M., Schwartz M., Haller R.G. (2009). Splice mutations preserve myophosphorylase activity that ameliorates the phenotype in McArdle disease. Brain.

[79] Le Guiner C., Servais L., Montus M., Larcher T., Fraysse B., Moullec S., Allais M., François V., Dutilleul M., Malerba A. (2017). Long-term microdystrophin gene therapy is effective in a canine model of Duchenne muscular dystrophy. Nat. Commun..

[80] Mack D.L., Poulard K., Goddard M.A., Latournerie V., Snyder J.M., Grange R.W., Elverman M.R., Denard J., Veron P., Buscara L. (2017). Systemic AAV8-Mediated Gene Therapy Drives Whole-Body Correction of Myotubular Myopathy in Dogs. Mol. Ther..

[81] Elverman M., Goddard M.A., Mack D., Snyder J.M., Lawlor M.W., Meng H., Beggs A., Buj-Bello A., Bs K.P., Marsh A.P. (2017). Long-term effects of systemic gene therapy in a canine model of myotubular myopathy. Muscle Nerve.

[82] Aguti S., Malerba A., Zhou H. (2018). The progress of AAV-mediated gene therapy in neuromuscular disorders. Expert Opin. Biol. Ther..

[83] Hermann T. (2007). Aminoglycoside antibiotics: Old drugs and new therapeutic approaches. Cell. Mol. Life Sci. CMLS.

[84] Du L., Damoiseaux R., Nahas S., Gao K., Hu H., Pollard J.M., Goldstine J., Jung M.E., Henning S.M., Bertoni C. (2009). Nonaminoglycoside compounds induce readthrough of nonsense mutations. J. Exp. Med..

[85] Birch K.E., Quinlivan R.M., Morris G.E. (2013). Cell models for McArdle disease and aminoglycoside-induced read-through of a premature termination codon. Neuromuscul. Disord. NMD.

[86] Dabrowski M., Bukowy-Bieryllo Z., Zietkiewicz E. (2015). Translational readthrough potential of natural termination codons in eucaryotes--The impact of RNA sequence. RNA Biol..

[87] Heinicke K., Dimitrov I.E., Romain N., Cheshkov S., Ren J., Malloy C.R., Haller R.G. (2014). Reproducibility and Absolute Quantification of Muscle Glycogen in Patients with Glycogen Storage Disease by 13C NMR Spectroscopy at 7 Tesla. PLoS ONE.

[88] Johnstone A.C., McSporran K.D., Kenny J.E., Anderson I.L., Macpherson G.R., Jolly R.D. (2004). Myophosphorylase deficiency (glycogen storage disease Type V) in a herd of Charolais cattle in New Zealand: Confirmation by PCR-RFLP testing. N. Z. Vet. J..

[89] Brooks E.D., Koeberl D.D. (2015). Large animal models and new therapies for glycogen storage disease. J. Inherit. Metab. Dis..

[90] Girard J., Ferre P., Pegorier J.P., Duee P.H. (1992). Adaptations of glucose and fatty acid metabolism during perinatal period and suckling-weaning transition. Physiol. Rev..

[91] De Castro M., Johnston J., Biesecker L. (2015). Determining the prevalence of McArdle disease from gene frequency by analysis of next-generation sequencing data. Genet. Med. Off. J. Am. Coll. Med. Genet..

